# Covid-19 pandemic policy monitor (COV-PPM) - European level tracking data of non-pharmaceutical interventions

**DOI:** 10.1016/j.dib.2021.107579

**Published:** 2021-11-16

**Authors:** Kayvan Bozorgmehr, Sven Rohleder, Stella Duwendag, Amir Mohsenpour, Victoria Saint, Andreas W. Gold, Sukhvir Kaur, Niklas Nutsch, Diogo Costa

**Affiliations:** a Department of Population Medicine and Health Services Research, School of Public Health, Bielefeld University, Bielefeld 33501, Germany; b Section for Health Equity Studies and Migration, Department of General Practice and Health Services Research, Heidelberg University Hospital, Germany

**Keywords:** Covid-19, SARS-Cov-2, Non-pharmaceutical interventions, Policy measures, Europe

## Abstract

The Covid-19 Pandemic Policy Monitor (COV-PPM) dataset prospectively documents non-pharmaceutical interventions (NPIs) taken to contain SARS-Cov-2 transmission across countries in EU27, EEA and UK. In Germany, measures have also been recorded at the federal state and, partially, at the district levels. NPIs implemented since January 2020 have been retrieved and updated weekly from March 2020, from official governments webpages, Ministries of Health, National (Public) Health Institutes or Administrations. NPI categories collected refer to restrictions, closures or changes in functioning implemented in 13 domains: public events (gatherings in indoor or outdoor spaces); public institutions (kindergartens, schools, universities); public spaces (shops, bars, restaurants); public transport (trains, buses, trams, metro); citizens movement/mobility (e.g. pedestrians, cars, ships); border closures (air, land or sea, all incoming travels, from high-risk regions, only non-nationals); measures to improve the healthcare system (e.g. human resources or technical reinforcement, redistribution, material or infrastructural); measures for risk/vulnerable groups (e.g. elderly, chronically ill, pregnant); economic measures (e.g. lay-off rules establishment, actions to avoid job-loss, tax relaxation); testing policies (e.g. testing criteria changes); nose and mouth protection rules, vaccination and others/miscellaneous measures.

## Specifications Table

**Table:** 

Subject	Health and Medical Sciences
Specific subject area	Public Health and Health Policy
Type of data	Tables and Figure
How the data were acquired	Primary sources for the recording of recommended or enforced non-pharmaceutical interventions (NPIs) were official websites of national health authorities (e.g. Ministries of Health, National Public Health Institutes and any specific websites managed by national governments to convey information about Covid-19), followed by national news websites. For German federal states and districts, information from health authorities of the corresponding geographical level was retrieved. For each NPI category the dates of implementation were coded with a textual description to contextualize each measure, and primary source saved (to pdf format). The categories established comprehensively cover several societal domains and regional resolutions.
Data format	Raw
Description of data collection	NPI categories refer to restrictions, closures or severe functioning changes implemented in 13 areas: public events, public institutions, public spaces, public transport, citizens' movement/mobility, border closures, measures to improve the healthcare system, measures for risk/vulnerable groups, economic measures, testing policies, nose and mouth protection rules, vaccinations, others. Categories are further detailed in Table 1.
Data source location	List of sources used to document non-pharmaceutical interventions being implemented is provided as supplementary material (Panel S1).
Data accessibility	The latest version of the dataset with NPIs implemented in European countries covered (prepared with R, updated until 14 December 2020) is available on the Harvard Dataverse Repository, including variable list description (codebook) (https://doi.org/10.7910/DVN/ANTOH7). Data visualizations and a description of the project are available online at https://www.uni-bielefeld.de/fakultaeten/gesundheitswissenschaften/ag/ag2/covid19.xml. Further queries and access to data covering the year 2021 and/ or to the German-level data can be made to the corresponding author: kayvan.bozorgmehr@uni-bielefeld.de

## Value of the Data

• COV-PPM dataset allows answering multiple research questions related to the impacts of specific measures on the SARS-Cov-2 pandemic across different geographical regions (32 European countries, including subnational data for Germany) or population subgroups and will also enable associations to be tested between higher-level characteristics (e.g., macro-economic indicators) and NPI effectiveness, accounting for other contextually relevant characteristics of the population.
• This dataset allows interested researchers to triangulate data, cross-validate NPI patterns and / or temporal sequences as collected by different initiatives and hence adds to the scientific process of consolidating knowledge on NPIs and their effectiveness in the European Region.
• The raw data is freely available from open repository and further queries (data covering 2021, German-level data) can be made to the responsible researchers, so that multiple approaches can be taken to analyze NPIs effectiveness. This is particularly important as there is no “gold standard” method for measuring NPIs as complex, multi-layered social response to the pandemic.

## 1 Data Description

The non-pharmaceutical intervention (NPI) categories covered in this dataset, comprehensively cover several societal domains and regional resolutions implemented in countries of EU-27, EEA, UK and across the 16 German federal states, as detailed in Table 1.

**Table 1:** Non-pharmaceutical interventions monitored by the COV-PPM in EU-27, EEA, UK and in the German federal states.

Domain of the policy measure	Description of information collected	Sub-categories specifying scope of measures (or regional resolution)
Public events	Major restrictions implemented are recorded, considering limits in the number of persons allowed for different types of public events or activities, in indoor or outdoor spaces. This may include conferences, amateur and professional collective sports, concerts, festivals, etc. Mandates to conduct health risk assessment for mass gatherings by authorities or collection of emergency contact details from participants was also recorded.	Not specified (code 1); More than 1000 persons (code 1a); Less than 1000 persons (code 1b); More than 50 persons (code 1c); Any type/number (code 1d);
Public institutions	Major restrictions or closures are considered and recorded for schools, universities, public services, etc. Restrictions to these institutions may include institution-wide intermediary measures aimed at physical distancing practices or enhancing hygiene measures (e.g. increasing in cleaning and disinfecting practices, in school buildings, classrooms, water and sanitation facilities), adjustment to space or set up of infrastructures (e.g. spacing of tables in schools), home office options for some or all staff. Records are kept if any criteria for restriction or closure pertains to type of institution, size or specific location.	Not specified (code 2); Single cities (code 2a); State level (code 2b); National (code 2c);
Public spaces	Restrictions and closures in public spaces are recorded (including shops, bars, swimming halls, gyms, restaurants, etc.), and any intermediary systematic measure taken aiming at keeping physical distancing and reducing transmission risk (e.g. cleaning and disinfecting measures). Adjustments to space, set up or infrastructures are also recorded (e.g. number of tables in restaurants, limitations in number of simultaneous visitors inside stores).	Not specified (code 3; Single cities (code 3a); State level (code 3b); National (code 3c);
Public transport	Measures affecting the transportation services and mobility of the public within a country, region or single city, regardless of the service being privately or publicly owned and run, were recorded and specified. This includes trains, buses, trams, metro transport. Restrictions to be recorded may include intermediary systematic measures (e.g. to keep physical distancing and reduce transmission risk), such as reduction in maximum number of passengers capacity, enhancing of hygiene and disinfecting measures and frequency, reduction of routes/frequency, measures to protect drivers (e.g. not opening of front doors in buses, or restriction in the sales of tickets by drivers).	Not specified (code 4); Single cities (code 4a); State level (code 4b); National (code 4c);
Movement/mobility	Restrictions in the movement of individuals by any means other than a public transport (e.g. pedestrians, by car, plane, or boat/ship), are recorded and specified regarding its application to entire national territories or specific regions, or single cities.	Not specified (code 5); Pedestrians (code 5a); Private cars (code 5b); Aviation national travel code 5c; Others (ships, trains, etc.) code 5d;
Travelling border closure	All border closures are recorded, specifying if restrictions applied to travelling by air, land or sea, across national borders. Quarantine and mandatory testing are also recorded. A distinction was also made regarding specific locations and criteria for which the restriction applied, namely if it applies to all travelers, to non-nationals only, to non-nationals from specific “high-risk areas”, or any other criteria used (or not specified).	Not specified (code 6); For non-nationals from high-risk regions (code 6a); for all non-nationals (code 6b); for all incoming travelers (code 6c);
Measures to improve the healthcare system	Any measures relating to Human Resources (HR) reinforcement in healthcare (e.g. re-activation of retired healthcare workers, re-location of healthcare workers according to increased needs, ease of recruitment procedures, paid child care for healthcare workers, transportation and accommodation specific for healthcare workers, etc.). A distinction is made to document technical measures taken, such as the shut-down or restriction of non-essential services (e.g. non-urgent surgery) in order to have additional capacity to treat Covid-19 patients, the creation/reinforcement of national telephone helplines (e.g. staffed by medical students). Material and infrastructure measures are considered, such as the setup of campaign hospitals settings (by adapting sports facilities, school pavilions, military tents, etc.), major acquisitions of material (e.g. ventilators, masks, visors, etc.).	Not specified (code 7); HR reinforcement or redistribution (code 7a); Technical reinforcement or redistribution (code 7b); Material infrastructural reinforcement or redistribution (code 7c);
Measures for risk/vulnerable groups	Any measure taken directed at the protection of vulnerable groups, specifically those aiming to protect older adults and the elderly (e.g. restriction of visitors in hospitals, nursing homes or facilities for the care of elderly), those with serious chronic conditions or who are immune-suppressed, or pregnant women. Measures specifically targeted at other socially vulnerable groups are considered, namely those directed at migrants (special focus to asylum seekers and refugees), people in homelessness situations (as defined by FEANTSA - European Federation of National Organizations working with the Homeless), and victims of domestic violence (and/or measures being specifically implemented to prevent different forms of domestic violence).	(code 8)
Economic measures	Major economic measures are recorded, namely all policies announcing non-suspension of services (e.g, gas, electricity, water or rent if there are delays in payment), or solidarity funds created for small businesses or independent workers, lay-off aid rules for employers, and other economic measures of different sorts that may be considered relevant in terms of their impact following the pandemic.	(code 9)
Miscellaneous	Other measures not fitting in the previous categories but deemed relevant by the team of researchers are recorded and if a certain type/category of measures repeatedly come up for one country or across several countries, a new category can be integrated even if a clear-cut hypothesis does not exist to include them as a variable with measurable impact in any outcome.	(code 10)
Testing policies	Changes in the policies about conducting tests for SARS-Cov-2 screening are recorded, as these are being indicated in authorities’ websites.	(code 11)
Mask policies	Mandatory or recommended use of mouth and nose protection (in specific settings, indoors or outdoors, etc), are recorded together with details of recommended context of utilization (e.g., age range, in public transports, schools, etc).	(code 12)
Vaccination	Strategy officially documented for vaccination (e.g. risk groups prioritized, specific programs implemented, channeled through ``normal'' health system or other specificity); whenever available, name of vaccine(s), producer/company, type of vaccine(s) are also documented.	Mandatory (code 13); Voluntary (code 13a); None (code 13b).

The proportion of days each measure was in place across the 32 European countries during the observation period from January 2020 until December 2020 are shown in Table 2, allowing to track patterns and differences between countries. For the 16 German federal states differences in specific domains can be tracked also within the country (Table S1, Supplementary Material).

**Table 2:** Proportion of days in which NPIs were in place in 32 countries (EU/EEA/UK) during the observation period (January 2020 to December 2020).

Vaccination measures are not included because their implementation started after the period considered for this table.

The analysis of textual elements and qualifiers (i.e., keywords identifying “restriction” or “withdrawal” added to each record) retrieved with each NPI description allows the creation of a subset of variables with disaggregation of broader selected categories: for example, in the case of public institutions, distinct variables were created to identify measures specifically aiming at (a) schools, (b) kindergartens, (c) universities/higher education, (d) gastronomy and (e) nightlife facilities. In the case of public events, this allows the creation of variables measuring changes to (a) public events indoors, (b) outdoors, and (c) in general.

**Fig. 1:**
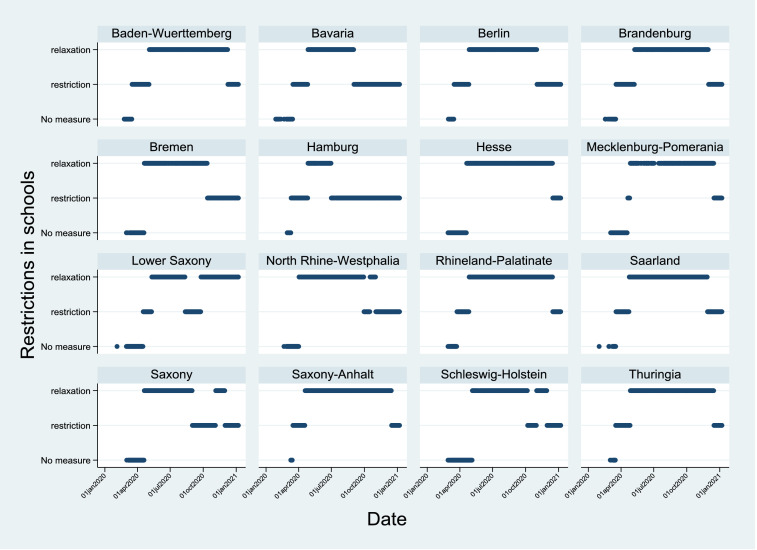
Measures implemented impacting schools across the German 16 federal states.

A detailed codebook of the resulting variables and description, from the disaggregation of measures impacting public institutions in the 16 federal states in Germany, are provided in the Supplementary Material (Table S2). Fig. 1 shows the result of NPIs related to schools, for the 16 German federal states. The same subset of variables can be generated for application at the EU-level.

## 2 Experimental Design, Materials and Methods

Since January 2020 and ongoing, the Covid-19 Pandemic Policy Monitor (COV-PPM) was established to record on a weekly basis, the non-pharmaceutical interventions (NPIs) taken in the EU27, EEA and UK (total of 32 countries). Additionally, data for Germany, where the research team is based, are being collected also at the federal state level (NUTS2) and partially at the district-level (NUTS3).

The categories of NPIs scoped were initially developed based on the knowledge available at the beginning of the pandemic with respect to measures being implemented in different countries and contexts, and complemented with the policies detailed in previous WHO reports and guidance (which are mainly based on the policies taken during the 2009 H1N1 influenza pandemic) [1]. The categories were progressively refined and adapted as the Covid-19 pandemic unfolded and different measures appeared (e.g. masks or mouth and nose protection, and vaccination rules and procedures).

The sources consulted for the recording of NPIs (recommended or enforced) were official websites of national health authorities (e.g. Ministries of Health, National Public Health Institutes and any specific websites managed by national governments to convey information about Covid-19), followed by national news websites. For German federal states and districts, information from health authorities of the corresponding geographical level was retrieved. Google translate was used for website translation whenever English versions of websites were not available. The team of researchers/assistants included German, French, Spanish and Portuguese speakers, that helped understand and document specific elements whenever translations provided dubious wording.

For each NPI category the team coded the exact dates of implementation and provided a textual description of the relevant information needed to contextualize each measure, saving the respective primary source (saved to pdf format, ensuring traceability of the collected information, since official health authorities websites, for example, are updated regularly).

The textual description added to each measure at the time of their coding, was preceded by selected keywords to allow qualification and recoding of NPIs according to their increasing or decreasing stringency. Specifically, for each point in time attached to the start, change or stop of each NPI category, a qualifier was added to identify and count if the coding refers to an additional “restriction”, “withdrawal” or “recommendation”. For the specific case of Masks utilization, two identifiers were added also to the categories where mouth and nose protection were additionally implemented as mandatory or recommended (e.g. enforcement of masks utilization in public transports, public institutions, public events).

To build our dataset, we recorded information in a long format with days as observation unit, coding each NPI from its starting to its ending date, as found in the corresponding sources. The text recorded following each keyword (i.e. after the start, ending or change of a measure indicated by the keywords “restriction”, withdrawal” or “recommendation”), provides a summarized description retrieved from the original source, that enables identification of the scope of application (for e.g. a withdrawal in public events: “*WITHDRAWAL: Wedding celebrations (after the actual wedding ceremony) are regarded as events without assigned and marked seats and can therefore take place with up to 100 people, regardless of whether they take place in a building or in the open-air area. From August 1st up to 200 people are allowed*.”

The team of researchers and assistants collecting the data, conducted frequent consensus meetings (weekly) and continuously updated the protocol for data collection according to changes also occurring throughout Europe, to encompass the nuances of increased or decreased stringency being implemented.

A Quality Assessment (QA) exercise was also conducted for the data gathered at the EU-level, and at the federal and district levels in Germany. The QA procedure consisted of a quantitative component and a qualitative one.

In the quantitative component, the team plotted the measures collected using interactive graphs, allowing a simple visualization of the aggregated data (example provided in supplementary material - Fig. S1). Based on these plots, the team searched for individual data points for each NPI to check for their accuracy and for potential non-plausible temporal sequences of NPIs (e.g. restrictions to public events or public transports coded for very short periods or with several interruptions in the time-series, measures coded before February 2020).

Following identification of potential coding mistakes visualized in the graphs, the individual country or federal state datasets were checked for the coding and a textual description recorded to assess whether an error was made in the documentation.

The qualitative component of the QA procedure consisted in analyzing the numerical coding and comments/textual elements saved for about 5–10% (around 2 weeks) of the total time sequence of each NPI and for each country, German federal state, and districts. The selection of the proportion of time to examine was based on the amount of comments/textual information registered (sequences with more frequent elements were selected but could vary for each NPI and for each geographical unit), and based on the available human resources. The numerical coding and textual information were then compared with the saved primary source to identify mismatches between the information provided in the sources and in the database. Two independent reviewers completed the QA process described for the German federal states, while most of the QA procedure at the EU level was conducted by a single reviewer. Any incongruencies found were then corrected.

The subnational dataset for Germany was recorded separately because of the federal nature of the country, which implies a high degree of autonomy for NPI implementation at subnational level. Although separate, the German subnational dataset was established following the same methodology and coding scheme used for European country-level, so that it can be directly merged into a single dataset.

A qualitative assessment of the text included for each subcategory is also being conducted, in order to establish even more fine-graded categories of measures that emerge across the different countries and German federal states, thus adding a layer of specificity to the analysis potential. Analysis of this qualitative information gathered, which is retrieved from countries’ official governmental sources, allows disentangling the change that occurred to the nature of some NPIs, for example, from complete closures to conditions under which specific public or private spaces can operate or host events (and which would have been aggregated as the same measure, otherwise). This also provides the needed flexibility to compare the data gathered with other existing public policy trackers for Covid-19, allowing for cross-validation.

This dataset shares some of the approaches and rationales of other trackers (e.g. with respect to covered domains of NPIs), but also differs from other efforts in its methodology [2], [3], [4], [5]. It aims to monitor NPIs prospectively, as they are being implemented, across 32 European nations, and with a special focus in Germany, where NPIs are being documented at the federal state and partially at district level. The German data collected (at the level of the 16 federal states) continues to be updated as measures are implemented and can be of great use to understand the effectiveness of NPIs taken at the local level, and establish further comparisons with the effectiveness measurable at the national and European levels. The federal nature of the German government implies a degree of autonomy for NPIs implementation, whose effectiveness to mitigate the diseases transmission can be tested if measures are available at such disaggregated levels. While continuation of recording of data at level of 401 German districts was desirable, the task could not be continued due to resource constraints.

## Ethics Statements

This project collects publicly available data, thus ethics approval was not needed.

## CRediT Author Statement

**Kayvan Bozorgmehr:** Conceptualization, Project administration, Data curation, Methodology, Writing - original draft, Writing – review & editing, Funding acquisition; **Sven Rohleder:** Data curation, Methodology, Validation, Writing – review & editing, Project administration, Formal analysis; **Stella Duwendag:** Data curation, Validation, Methodology, Writing – review & editing, Project administration, Formal analysis; **Amir Mohsenpour:** Data curation, Methodology, Writing – review & editing; **Victoria Saint:** Data curation, Methodology, Writing – review & editing; **Andreas W. Gold:** Data curation, Methodology, Writing – review & editing; **Sukhvir Kaur:** Data curation, Methodology, Validation, Writing – review & editing; **Niklas Nutsch:** Data curation, Methodology, Writing – review & editing; **Diogo Costa:** Data curation, Methodology, Validation, Writing - original draft, Writing – review & editing, Project administration, Formal analysis.

## Declaration of Competing Interest

The authors declare that they have no known competing financial interests or personal relationships that could have appeared to influence the work reported in this paper.

## Appendix Supplementary materials

Image, application 1
