# Supplementary material for: Covid-19 pandemic policy monitor (COV-PPM) - European level tracking data of non-pharmaceutical interventions
Source: Data Brief. 2021 Nov 16;39:107579. doi: 10.1016/j.dib.2021.107579 (PMC8592638; doi:10.1016/j.dib.2021.107579)
Supplement: Supplementary file 1 [file mmc1.docx]

**Supplementary Material**

Table S1. Proportion of days in which NPIs were in place in 16 German federal states during the observation period (January 2020 to December 2020)

| **Federal states** | **Public events** | **Public institutions** | **Public spaces** | **Public transport** | **Movement/**  **mobility** | **Border closures** | **Healthcare system measures** | **Vulnerable groups** | **Economic** | **Miscellaneous** | **Testing policies** | **Masks** |
| --- | --- | --- | --- | --- | --- | --- | --- | --- | --- | --- | --- | --- |
| Saxony-Anhalt | 99,7% | 98,3% | 97,6% | 85,4% | 96,2% | 97,2% | 16,0% | 97,6% | 17,7% | 0,0% | 76,4% | 85,1% |
| Saxony | 98,3% | 94,9% | 95,2% | 95,2% | 93,9% | 87,8% | 94,6% | 94,6% | 29,6% | 31,6% | 73,8% | 84,4% |
| Bremen | 98,0% | 97,6% | 95,9% | 82,3% | 93,5% | 96,3% | 90,1% | 95,9% | 0,0% | 25,5% | 4,1% | 82,3% |
| Saarland | 97,9% | 96,9% | 96,9% | 95,5% | 94,4% | 65,7% | 96,9% | 96,9% | 0,3% | 36,0% | 69,9% | 79,0% |
| Thuringia | 97,6% | 96,9% | 96,6% | 84,1% | 93,9% | 88,8% | 97,3% | 96,6% | 13,2% | 31,5% | 80,3% | 84,1% |
| Mecklenburg-Pomerania | 97,3% | 95,7% | 95,7% | 82,9% | 95,7% | 95,7% | 86,8% | 95,7% | 57,4% | 76,4% | 32,6% | 68,6% |
| Schleswig-Holstein | 97,3% | 95,2% | 93,9% | 95,2% | 94,5% | 92,8% | 87,7% | 83,3% | 25,9% | 32,4% | 95,2% | 80,2% |
| Berlin | 96,1% | 95,4% | 96,1% | 81,6% | 93,4% | 93,1% | 96,1% | 96,1% | 0,0% | 0,3% | 5,3% | 83,2% |
| Hamburg | 96,0% | 96,7% | 96,7% | 94,7% | 93,1% | 0,0% | 0,0% | 94,4% | 0,3% | 0,3% | 46,2% | 81,2% |
| Hesse | 95,8% | 95,1% | 94,5% | 92,8% | 96,1% | 42,0% | 96,1% | 96,1% | 56,7% | 0,7% | 0,3% | 83,7% |
| North Rhine-Westphalia | 95,5% | 93,6% | 93,6% | 92,9% | 85,9% | 93,6% | 94,6% | 94,6% | 61,9% | 0,0% | 87,8% | 81,4% |
| Brandenburg | 95,0% | 95,0% | 95,0% | 77,9% | 95,3% | 65,4% | 17,4% | 95,0% | 0,3% | 0,0% | 5,7% | 77,9% |
| Rhineland-Palatinate | 93,4% | 92,5% | 93,4% | 92,5% | 86,9% | 53,4% | 92,5% | 92,5% | 0,3% | 57,7% | 5,6% | 83,6% |
| Baden-Württemberg | 93,0% | 93,0% | 93,0% | 79,9% | 91,7% | 92,0% | 0,0% | 93,0% | 0,0% | 59,4% | 80,2% | 79,9% |
| Bavaria | 86,6% | 91,3% | 89,7% | 86,6% | 89,7% | 0,0% | 91,3% | 89,7% | 90,0% | 37,2% | 96,9% | 80,3% |
| Lower Saxony | 83,4% | 84,4% | 90,9% | 90,9% | 90,9% | 88,6% | 88,3% | 83,8% | 48,4% | 56,2% | 0,0% | 76,6% |

Vaccination measures are not included because their implementation started after the period considered for this table.

| **Panel S1**  **List of sources used to document non-pharmaceutical interventions being implemented in COV-PPM**  **Countries**  Austria: <https://www.sozialministerium.at/Informationen-zum-Coronavirus/Coronavirus---Haeufig-gestellte-Fragen.html>  Belgium: <https://www.info-coronavirus.be/en/> <https://www.vlaanderen.be/gezondheid-en-welzijn/gezondheid/gezondheid-en-preventie-tijdens-de-coronacrisis> <https://coronavirus.brussels/index.php/en/> <http://www.ejustice.just.fgov.be/cgi/welcome.pl> [https://www.wallonie.be/fr/actualites/coronavirus-COVid-19-mesures-regionales](https://www.wallonie.be/fr/actualites/coronavirus-covid-19-mesures-regionales)  Bulgaria: <https://www.mh.government.bg/bg/novini/aktualno/>  Croatia: <https://www.koronavirus.hr/> <https://www.koronavirus.hr/najnovije/34>  Cyprus: <https://www.pio.gov.cy/coronavirus/>  Czech Republic: <https://koronavirus.mzcr.cz/> <https://www.vlada.cz/scripts/detail.php?pgid=103&conn=3151&pg=1> <https://www.vlada.cz/cz/epidemie-koronaviru/dulezite-informace/mimoradna-opatreni-_-co-aktualne-plati-180234/>  Denmark: <https://um.dk/en/> <https://coronasmitte.dk/en> <https://www.sst.dk/en/English> <https://www.ssi.dk/aktuelt/sygdomsudbrud/coronavirus> <https://en.ssi.dk/> <https://coronasmitte.dk/en/overview>  Estonia: <https://www.sm.ee/et> <https://www.kriis.ee/et> <https://www.valitsus.ee/et/eriolukord-eestis#meetmed> <https://kkk.kriis.ee/et/kkk/fookuses-koroonakriisist-jarkjargult-valjatulek>  Finland: [https://thl.fi/en/web/infectious-diseases/what-s-new/coronavirus-COVid-19-latest-updates](https://thl.fi/en/web/infectious-diseases/what-s-new/coronavirus-covid-19-latest-updates) <https://valtioneuvosto.fi/en/information-on-coronavirus> <https://stm.fi/>  France: <https://www.gouvernement.fr/info-coronavirus> <https://solidarites-sante.gouv.fr/> <https://www.santepubliquefrance.fr/>  Germany: <https://www.bundesregierung.de/breg-de/themen/coronavirus>  <https://www.bundesgesundheitsministerium.de/service/gesetze-und-verordnungen.html>  Greece: <https://www.moh.gov.gr/articles/ministry/grafeio-typoy/press-releases> [https://www.moh.gov.gr/articles/health/dieythynsh-dhmosias-ygieinhs/metra-prolhpshs-enanti-koronoioy-sars-COV-2/](https://www.moh.gov.gr/articles/health/dieythynsh-dhmosias-ygieinhs/metra-prolhpshs-enanti-koronoioy-sars-cov-2/) <https://eody.gov.gr/category/epikairotita/> <https://www.civilprotection.gr/el> https://COVid19.gov.gr/category/proliptika-metra-gia-tin-pandimia/ <https://www.civilprotection.gr/el/announcements> [https://COVid19.gov.gr/](https://covid19.gov.gr/)  Hungary: <http://abouthungary.hu/blog/pm-orban-announced-extension-of-economy-protection-action-plan/>  Iceland: <https://www.government.is/news/> <https://www.landlaeknir.is/koronaveira/thad-sem-thu-tharft-ad-vita/> [https://www.COVid.is/](https://www.covid.is/)  Ireland: [https://www.gov.ie/en/news/](https://www.gov.ie/en/news/58bc8b-taoiseach-announces-roadmap-for-reopening-society-and-business-and-u/?referrer=http://www.gov.ie/roadmap/)  Italy: [http://www.salute.gov.it/portale/nuovocoronavirus/](http://www.salute.gov.it/portale/nuovocoronavirus/dettaglioContenutiNuovoCoronavirus.jsp?lingua=english&id=5367&area=nuovoCoronavirus&menu=vuoto) <https://www.gazzettaufficiale.it/dettaglioArea/12> <http://www.salute.gov.it/portale/news/p3_2.html>  Latvia: [https://www.mk.gov.lv/lv/COVid-19](https://www.mk.gov.lv/lv/covid-19) [https://COVid19.gov.lv/index.php/en](https://covid19.gov.lv/index.php/en) <https://www.vm.gov.lv/lv>  Liechtenstein: <https://www.regierung.li/coronavirus> <https://www.llv.li/medienmitteilungen> <https://hebensorg.li/> [https://tourismus.li/unser-land/ueber-liechtenstein/aktuelle-informationen-zu-coronaCOVid-19-im-fuerstentum-liechtenstein/](https://tourismus.li/unser-land/ueber-liechtenstein/aktuelle-informationen-zu-coronacovid-19-im-fuerstentum-liechtenstein/) <https://www.wko.at/service/aussenwirtschaft/coronavirus-newsticker-schweiz.html> <https://www.liechtenstein.li/land-und-leute/gesellschaft/gesundheitswesen/corona-virus/>  Lithuania: <https://lrv.lt/en/news> <https://koronastop.lrv.lt/en/> [https://urm.lt/default/en/important-COVid19](https://urm.lt/default/en/important-covid19)  Luxembourg: [https://COVid19.public.lu/de/communications-officielles.html](https://covid19.public.lu/de/communications-officielles.html) <https://gouvernement.lu/fr/actualites/toutes_actualites.html> [https://data.public.lu/en/datasets/COVid-19-rapports-hebdomadaires/](https://data.public.lu/en/datasets/covid-19-rapports-hebdomadaires/)  Malta: <https://deputyprimeminister.gov.mt/en/Pages/health.aspx>  Netherlands: [https://www.rijksoverheid.nl/onderwerpen/coronavirus-COVid-19/nieuws](https://www.rijksoverheid.nl/onderwerpen/coronavirus-covid-19/nieuws)  Norway: <https://www.regjeringen.no/en/whatsnew/finn-aktuelt2/id2415244/?topic=2692388> [https://www.regjeringen.no/en/topics/koronavirus-COVid-19/timeline-for-news-from-norwegian-ministries-about-the-coronavirus-disease-COVid-19/id2692402/](https://www.regjeringen.no/en/topics/koronavirus-covid-19/timeline-for-news-from-norwegian-ministries-about-the-coronavirus-disease-covid-19/id2692402/) <https://www.fhi.no/en/> <http://www.norwaynews.com/>  Poland: <https://www.gov.pl/web/coronavirus> <https://www.gov.pl/web/koronawirus/dzialania-rzadu> <https://www.gov.pl/web/coronavirus/travel> <https://www.gov.pl/web/coronavirus/temporary-limitations>  Portugal: [https://dre.pt/legislacao-COVid-19-upo](https://dre.pt/legislacao-covid-19-upo) [https://COVid19estamoson.gov.pt/medidas-excecionais/](https://covid19estamoson.gov.pt/medidas-excecionais/)  Romania: <https://stirioficiale.ro/>  Slovakia: [https://www.korona.gov.sk/COVid-19-usmernenia-obcania.php](https://www.korona.gov.sk/covid-19-usmernenia-obcania.php) [https://www.standardnepostupy.sk/klinicky-protokol-spdtp-klinicky-manazment-podozrivych-a-potvrdenych-pripadov-COVid-19/](https://www.standardnepostupy.sk/klinicky-protokol-spdtp-klinicky-manazment-podozrivych-a-potvrdenych-pripadov-covid-19/)  Slovenia: <https://www.gov.si/en/news?date=&nrOfItems=20&start=0&tag%5B0%5D=554>  Spain: <https://administracion.gob.es/> <https://www.lamoncloa.gob.es/lang/en/Paginas/index.aspx>  Sweden: [https://www.folkhalsomyndigheten.se/smittskydd-beredskap/utbrott/aktuella-utbrott/COVid-19/](https://www.folkhalsomyndigheten.se/smittskydd-beredskap/utbrott/aktuella-utbrott/covid-19/) [https://www.government.se/government-policy/the-governments-work-in-response-to-the-virus-responsible-for-COVid-19/](https://www.government.se/government-policy/the-governments-work-in-response-to-the-virus-responsible-for-covid-19/)  Switzerland: <https://www.bag.admin.ch/bag/en/home.html> <https://www.admin.ch/gov/en/start/documentation/media-releases.html?dyn_startDate=01.01.2015&dyn_organization=1>  UK: <https://www.gov.uk/search/news-and-communications> <https://www.england.nhs.uk/coronavirus/> <https://www.wikiwand.com/en/COVID-19_pandemic_in_the_United_Kingdom>  **German federal states**  Baden-Württemberg: <https://www.baden-wuerttemberg.de/de/service/aktuelle-infos-zu-corona/aktuelle-corona-verordnung-des-landes-baden-wuerttemberg/>  Bavaria: <https://www.stmgp.bayern.de/coronavirus/rechtsgrundlagen/>  Berlin: <https://www.berlin.de/corona/massnahmen/verordnung/>  Brandenburg: <https://www.landesrecht.brandenburg.de/dislservice/public/index.jsp>; <https://brandenburg-impft.de/bb-impft/de/aktuelles/>; <https://mik.brandenburg.de/mik/de/start/service/presse/pressemitteilungen/>  Bremen: <https://www.gesetzblatt.bremen.de>; <https://www.amtliche-bekanntmachungen.bremen.de>  Hamburg: <https://www.luewu.de/gvbl/>; <https://www.hamburg.de/allgemeinverfuegungen/>  Hesse: <https://www.hessen.de/fuer-buerger/corona-hessen/verordnungen-und-allgemeinverfuegungen>; https://www.hessen.de/fuer-buerger/corona-in-hessen/interviews-reden-und-mehr/corona-massnahmen-der-landesregierung-seit-dezember-2020  Lower Saxony: <https://www.niedersachsen.de/Coronavirus/vorschriften/vorschriften-der-landesregierung-185856.html>; <https://www.niedersachsen.de/Coronavirus>; <https://www.niedersachsen.de/Coronavirus/aktuelle-presseinformationen-186247.html#aeltere_Meldungen>  Mecklenburg-Vorpommern: <https://www.regierung-mv.de/corona/Verordnungen-und-Dokumente/>; <https://www.regierung-mv.de/corona/#wichtige%20Dokumente>; <https://www.regierung-mv.de/Aktuell>; <https://www.lagus.mv-regierung.de/Gesundheit/InfektionsschutzPraevention/Impfen-Corona-Pandemie/>  North-Rhine-Westphalia: <https://www.mags.nrw/coronavirus-rechtlicheregelungen-nrw>; <https://www.land.nrw/nl/node/23375>  Rhineland-Palatinate: <https://corona.rlp.de/de/startseite/>; <https://corona.rlp.de/de/service/rechtsgrundlagen/>  Saarland: <https://www.saarland.de/DE/portale/corona/service/rechtsverordnung-massnahmen/rechtsverordnung-massnahmen_node.html>  Saxony: <https://www.coronavirus.sachsen.de/amtliche-bekanntmachungen.html>; <https://www.coronavirus.sachsen.de/index.html>; <https://www.coronavirus.sachsen.de/newsroom-4155.html>  Saxony-Anhalt: <https://ms.sachsen-anhalt.de/themen/gesundheit/aktuell/coronavirus/>  Schleswig-Holstein: <https://www.schleswig-holstein.de/DE/Landesregierung/Themen/GesundheitVerbraucherschutz/Coronavirus/coronavirus.html>; <https://www.schleswig-holstein.de/DE/Schwerpunkte/Coronavirus/_documents/teaser_erlasse.html;jsessionid=F274B822369590071BEF2060DEBF64AE.delivery1-replication>; <https://www.schleswig-holstein.de/DE/Schwerpunkte/Coronavirus/Presse/PI/pressemitteilungen_node.html>  Thuringia: [https://www.tmasgff.de/COVid-19/rechtsgrundlage](https://www.tmasgff.de/covid-19/rechtsgrundlage) <https://corona.thueringen.de/> |
| --- |





Figure S1. Example of Horizontal display bar chart of temporal sequence of NPIs in different domains by country in EU-27, EEA, UK, 01/2020-01/2021, categorical data gathered.

Table S2. Codebook with the variables created to disaggregate information about major restrictions to the functioning of public institutions identified in the scope of COV-PPM, in Germany.

| **Category** | **Item** | **Variable** | **Variable value** | **Description** |
| --- | --- | --- | --- | --- |
| Public institutions | **School** | school | 0 = no measure 1 = restricted measure 2 = relaxed measure 3 = both measures in place | General restricted and relaxed measures in schools.  "3 = both measures in place" means that both restricted and relaxed measures are present in schools at the same time. |
|  |  | school_prim | 0 = no measure 1 = restricted measure 2 = relaxed measure 3 = both measures in place | General restricted and relaxed measures in primary schools.  "3 = both measures in place" means that both restricted and relaxed measures are present at the same time. |
|  |  | school_sec | 0 = no measure 1 = restricted measure 2 = relaxed measure 3 = both measures in place | General restricted and relaxed measures in secondary schools.  "3 = both measures in place" means that both restricted and relaxed measures are present at the same time. |
|  |  | school_res | 0 = no measure 1 + n = cumulative number of restricted measures | Cumulative number of restricted school measures over time. The first restriction is given the value 1. All subsequent new restrictions become 1 + n number of previous restrictions. |
|  |  | school_mit | 0 = no measure 1 + n = cumulative number of relaxed measures | Cumulative number of relaxed school measures over time. The first relaxation is given the value 1. All subsequent new relaxations come 1 + n number of previous relaxations. |
|  |  | school_res_mit | 1 = zero point | Interval scaled variable measuring the change in present restricted and relaxed measures.  If there is a restriction, the value is increased by 0.01 points or decreased by this value if there is a relaxation. |
|  |  | school_closure | 0 = no measure in place 1 = closure 2 = opening | Categorical measure of school closures. |
|  |  | school_prim_closure | 0 = no measure in place 1 = closure 2 = opening | Categorical measure of primary school closures. |
|  |  | school_sec_closure | 0 = no measure in place 1 = closure 2 = opening | Categorical measure of secondary school closures. |
| Public institutions | **Kindergarten** | kita | 0 = no measure 1 = restricted measure 2 = relaxed measure 3 = both measures in place | General restricted and relaxed measures in kindergarten and nursery schools.  "3 = both measures in place" means that both restricted and relaxed measures are present at the same time. |
|  |  | kita_res | 0 = no measure 1 + n = cumulative number of restricted measures | Cumulative number of restricted kindergarten and nursery school measures over time. The first restriction is given the value 1. All subsequent new restrictions become 1 + n number of previous restrictions. |
|  |  | kita_mit | 0 = no measure 1 + n = cumulative number of relaxed measures | Cumulative number of relaxed kindergarten and nursery school measures over time. The first relaxation is given the value 1. All subsequent new relaxations become 1 + n number of previous relaxations. |
|  |  | kita_res_mit | 1 = zero point | Interval scaled variable measuring the change in present restricted and relaxed measures.  If there is a restriction, the value is increased by 0.01 points or decreased by this value if there is a relaxation. |
|  |  | kita_closure | 0 = no measure in place 1 = closure 2 = opening | Categorical measure of kindergarten and nursery school closures. |
| Public institutions | **University/ higher eduction** | university | 0 = no measure 1 = restricted measure 2 = relaxed measure 3 = both measures in place | General restricted and relaxed measures in universities and facilities of higher education.  "3 = both measures in place" means that both restricted and relaxed measures are present at the same time. |
|  |  | university_res | 0 = no measure 1 + n = cumulative number of restricted measures | Cumulative number of restricted universities and facilities of higher education measures over time. The first restriction is given the value 1. All subsequent new restrictions become 1 + n number of previous restrictions. |
|  |  | university_mit | 0 = no measure 1 + n = cumulative number of relaxed measure | Cumulative number of relaxed universities and facilities of higher education measures over time. The first relaxation is given the value 1. All subsequent new relaxations become 1 + n number of previous relaxations. |
|  |  | university_res_mit | 1 = zero point | Interval scaled variable measuring the change in present restricted and relaxed measures.  If there is a restriction, the value is increased by 0.01 points or decreased by this value if there is a relaxation. |
|  |  | university_closure | 0 = no measure in place 1 = closure 2 = opening | Categorical measure of universities and facilities of higher education closures. |
| Public institutions &  Public spaces | **Cultural and  educational institutions** | culture | 0 = no measure 1 = restricted measure 2 = relaxed measure 3 = both measures in place | General restricted and relaxed measures in cultural and educational institutions.  "3 = both measures in place" means that both restricted and relaxed measures are present at the same time. |
|  |  | culture_res | 0 = no measure 1 + n = cumulative number of restricted measures | Cumulative number of restricted cultural and educational institutions measures over time. The first restriction is given the value 1. All subsequent new restrictions become 1 + n number of previous restrictions. |
|  |  | culture_mit | 0 = no measure 1 + n = cumulative number of relaxed measures | Cumulative number of relaxed cultural and educational institutions measures over time. The first relaxation is given the value 1. All subsequent new relaxations become 1 + n number of previous relaxations. |
|  |  | culture_res_mit | 1 = zero point | Interval scaled variable measuring the change in present restricted and relaxed measures.  If there is a restriction, the value is increased by 0.01 points or decreased by this value if there is a relaxation. |
|  |  | culture_closure | 0 = no measure in place 1 = closure 2 = opening | Categorical measure of cultural and educational institutions closures. |
| Public institutions | **Workplace restrictions** | workplace | 0 = no measure 1 = restricted measure 2 = relaxed measure 3 = both measures in place | General restricted and relaxed measures in workplace.  "3 = both measures in place" means that both restricted and relaxed measures are present at the same time. |
|  |  | workplace_res | 0 = no measure 1 + n = cumulative number of restricted measures | Cumulative number of restricted workplace measures over time. The first restriction is given the value 1. All subsequent new restrictions become 1 + n number of previous restrictions. |
|  |  | workplace_mit | 0 = no measure 1 + n = cumulative number of relaxed measures | Cumulative number of relaxed workplace measures over time. The first relaxation is given the value 1. All subsequent new relaxations become 1 + n number of previous relaxations. |
|  |  | workplace_res_mit | 1 = zero point | Interval scaled variable measuring the change in present restricted and relaxed measures.  If there is a restriction, the value is increased by 0.01 points or decreased by this value if there is a relaxation. |
